# Sunitinib as salvage treatment including potent anti-tumor activity in carcinomatous ulcers for patients with multidrug-resistant metastatic breast cancer

**DOI:** 10.18632/oncotarget.11082

**Published:** 2016-08-05

**Authors:** Bing Sun, Xin Zhao, Lijuan Ding, Xiangying Meng, Santai Song, Shikai Wu

**Affiliations:** ^1^ Department of Radiotherapy, Affiliated Hospital of Academy of Military Medical Sciences, Beijing, 100071, China; ^2^ Department of Radiotherapy, Department of Breast Cancer, Affiliated Hospital of Academy of Military Medical Sciences, Beijing, 100071, China

**Keywords:** breast cancer, multidrug resistance, sunitinib, salvage treatment

## Abstract

**Objective:**

To evaluate the efficacy and safety of single-agent sunitinib as salvage treatment in Chinese patients with multidrug-resistant metastatic breast cancer (MBC).

**Results:**

37 patients were enrolled with median age of 48 years. 17 had hormone receptor (HR)-positive tumors, 7 had HER2-positive tumors, and 10 had triple-negative tumors. Among 32 evaluable patients with follow-up, 6 (18.8%) achieved partial response, 14 (43.8%) achieved stable disease, and 11 (34.4%) exhibited tumor shrinkage. The response rate in 9 patients with carcinomatous ulcers was 77.8%. The median progression free survival (PFS) was 8.6 weeks. Patients with a better response had improved overall survival and PFS relative to patients with a worse response (*p* = 0.007, *p* < 0.001). Compared with HR-negative tumor, HR-positive tumor had significantly better response to sunitinib (*p* = 0.035). The most frequent non-hematologic adverse events were fatigue (82.8%) and hypertension (34.5%). Grade 3/4 hematologic toxicity included neutropenia (82.8%) and thrombocytopenia (79.3%). There was no correlation between the clinical response and IHC findings.

**Materials and Methods:**

Patients with MBC who were resistant to multiple salvage regimens (≥ 3 previous chemotherapy lines) were enrolled to receive sunitinib monotherapy. Dosage adjustment was allowed depending on adverse events. 14 patients underwent immunohistochemistry (IHC) testing for VEGF, PDGFR, EGFR and c-KIT.

**Conclusions:**

Sunitinib salvage treatment provided modest antitumor effect to patients with refractory multidrug-resistant MBC, especially to those with troublesome carcinomatous ulcers. The treatment-related adverse events of sunitinib were manageable through dosage adjustment.

## INTRODUCTION

Medical treatment is the major treatment option for patients with metastatic breast cancer (MBC). It is difficult to control tumor and extend survival, even with all possible available modalities. Therefore, finding an effective treatment for such patients is urgent. In recent years, an increasing number of molecular targeted agents have offered clinicians new options.

Sunitinib malate is an oral small-molecule, multitargeted tyrosine kinase inhibitor that exerts both antitumor effects and antiangiogenic actions via inhibition of platelet-derived growth factor receptors (PDGFR), vascular endothelial growth factor receptors (VEGFR), stem cell factor receptor (c-KIT), FMS-like tyrosine kinase-3 receptor (FLT3), the receptor for macrophage colony-stimulating factor (CSF-1R), and glial cell-line-derived neurotrophic factor receptor (RET) [1]. The efficacy of sunitinib has been demonstrated in patients with gastrointestinal stromal tumors (GIST) and renal cell carcinoma (RCC) [2–4].

Several studies confirmed that PDGF signaling pathway implicated in the pathogenesis of breast cancer, and angiogenesis was inhibited in breast cancer xenografts by sunitinib [5, 6]. In view of these preclinical evidences, sunitinib was used in phase I/II clinical trials and has demonstrated modest single-agent effect [7–10]. In subsequent phase III clinical studies, sunitinib has failed to improve survival of MBC compared with other standard regimens [11–14]. However, its efficacy in Asian MBC patients has not yet been reported. Furthermore, there is either definitive treatment strategy or effective chemotherapy regimen available for multidrug-resistant MBC. Therefore, the present study was performed to assess the efficacy and safety of sunitinib monotherapy for Chinese patients with refractory heavily pretreated MBC. We hypothesized that inhibition of multiple signaling pathways would yield an efficacy benefit and tumor control in this specific population.

## RESULTS

### Baseline characteristics

Thirty-seven Chinese MBC patients were enrolled with median age of 48 years (range 27–70). The median previous salvage chemotherapy lines was 7 (range 3–17). 5 patients were unable to be evaluated due to the loss of follow-up. The clinical characteristics of 32 evaluable patients are shown on Table 1. 17 patients had HR-positive (estrogen receptor (ER) and/or progesterone receptor (PR) positive) tumors, 7 patients had HER2-positive tumors, and 10 patients had triple-negative tumors. All patients had multidrug-resistant disease and were resistant to taxane and anthracycline. 19 (59.4%) patients were resistant to endocrine therapy. Of note, 9 patients had carcinomatous ulcers.

**Table 1:** Clinical characteristics and responses of sunitinib therapy in 32 evaluable patients with follow-up

Characteristic	*n*	CR	PR	SD (improved)^a^	SD	PD	Response rate^b^ (%)	*P*-value
Age of onset								0.811
≥ 50 years	10	0	2	3	2	3	50.0%	
< 50 years	22	0	4	8	1	9	54.5%	
KPS score:								0.529
70–80	11	0	2	3	0	6	45.5%	
≥ 90	21	0	4	8	3	6	57.1%	
Endocrine therapy								0.784
Previous endocrine therapy	20	0	3	8	2	7	55.0%	
No previous endocrine therapy	12	0	3	3	1	5	50.0%	
Number of prior chemotherapy lines								0.892
≥ 7	17	0	4	5	3	5	52.9%	
< 7	15	0	2	6	0	7	53.3%	
Metastatic sites								0.574
Skin and soft tissue (e.g. lymph node, etc.)	23	0	5	8	1	9	56.5%	
Lung	16	0	4	5	2	5	56.3%	
Liver	13	0	1	3	1	8	30.8%	
Brain	3	0	0	2	0	1	66.7%	
Bone	17	0	2	7	2	6	52.9%	
Number of metastatic sites								0.927
Single	2	0	0	1	1	0	50.0%	
Multiple	30	0	6	10	2	12	53.3%	
Receptor status of primary tumor								
HR (+)	17	0	3	9	0	5	70.6%	0.035
HR (−)	15	0	3	2	3	7	33.3%	
HER-2 (+)	7	0	1	2	3	1	42.9%	0.678
HER-2 (−)	25	0	5	9	0	11	56.0%	

a SD (improved) = patients with stable disease who exhibited tumor shrinkage (0–29.9% decrease in the sum of the longest diameters of target lesions compared with baseline).

b Response rate = CR + PR + SD (improved).

Abbreviations: CR, complete response; HR, hormone receptor; HER-2, human epidermal growth factor receptor-2; KPS, Karnofsky performance status; PD, progressive disease; PR, partial response; SD, stable disease.

### Clinical efficacy

**Figure 1:**
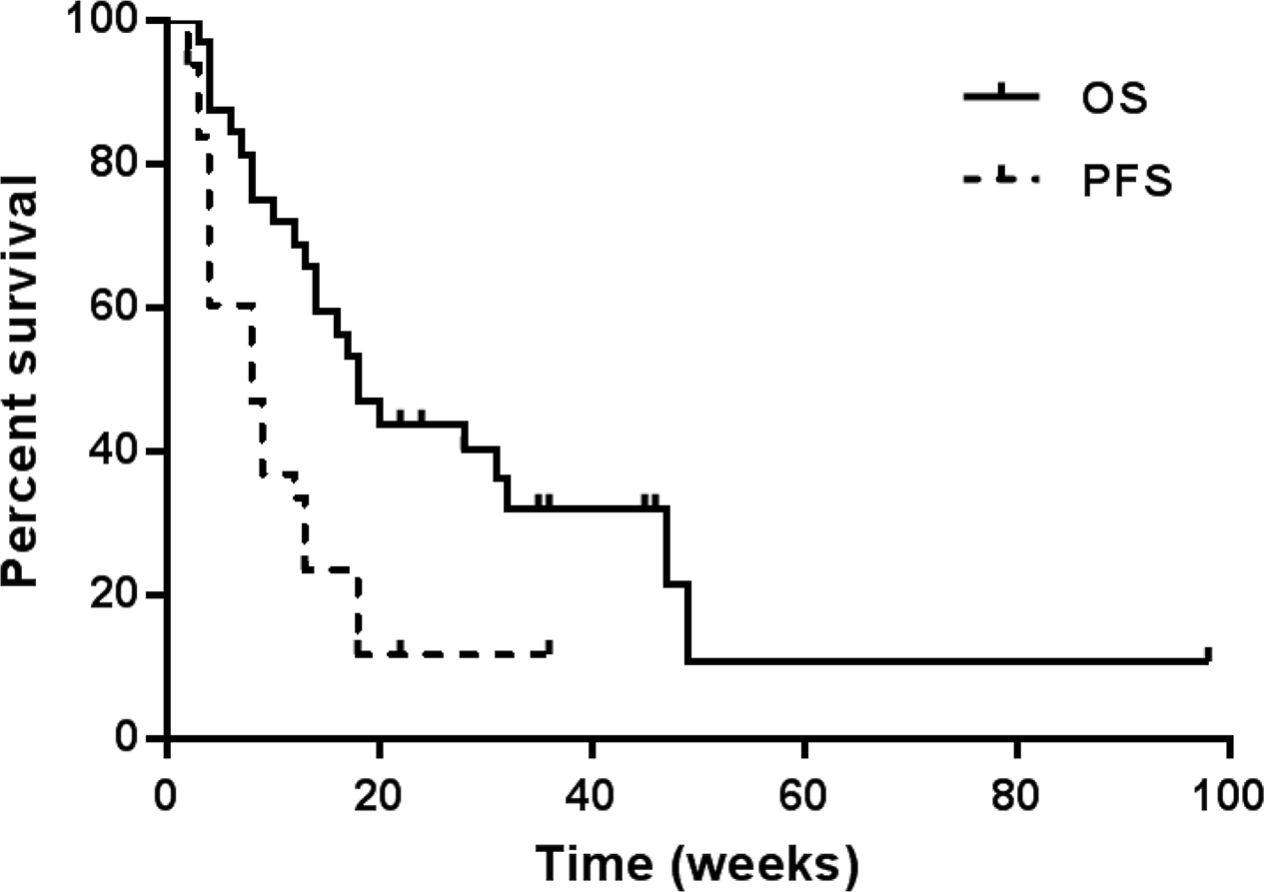
Progression-free survival (PFS) and overall survival (OS) of sunitinib monotherapy in 32 patients with metastatic breast cancer

**Figure 2:**
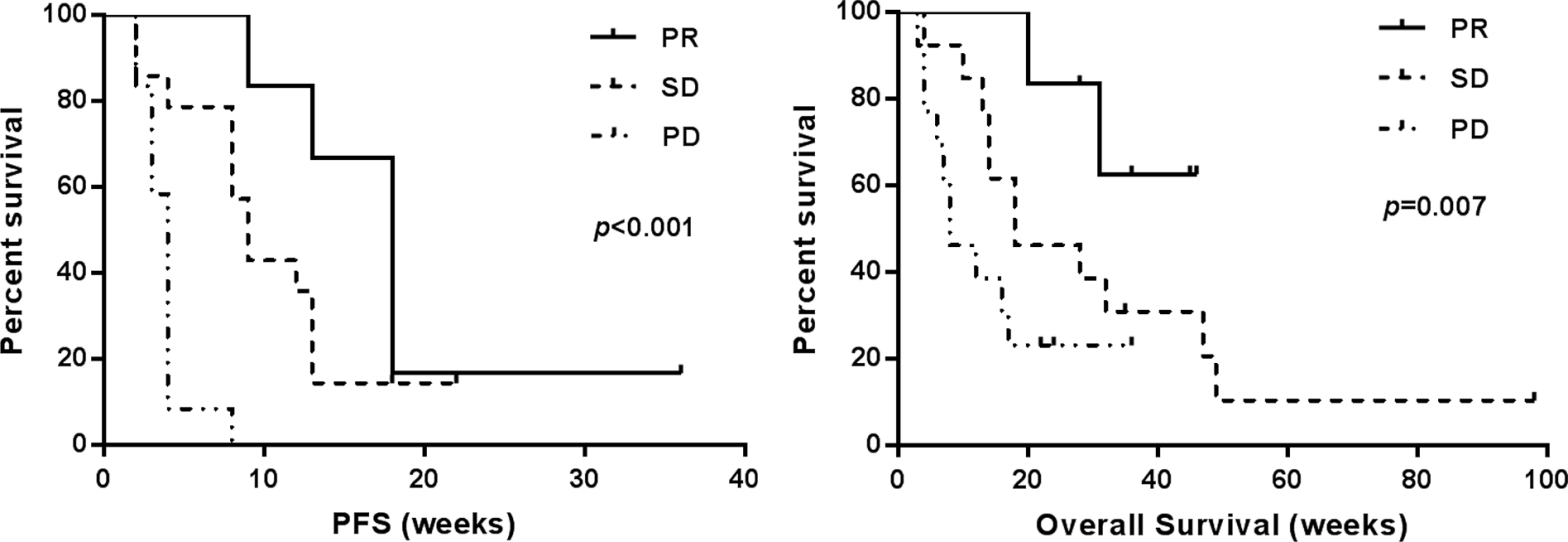
Progression-free survival (PFS) and overall survival of sunitinib monotherapyin 32 patients with metastatic breast cancer by response

After a median follow-up of 30 weeks (range 2–98 weeks), 32 patients died and 5 were lost to follow-up. Among the 32 evaluable patients, 6 (18.8%) achieved PR, 14 (43.8%) achieved SD (5 exhibited tumor shrinkage), and 12 (37.5%) confirmed PD. A total of 11 (34.4%) patients exhibited tumor shrinkage (Table 1). Patients with HR-positive tumor had significantly better clinical response (PR + SD improved) to sunitinib compared to patients with HR-negative tumor (*p* = 0.035). The median PFS and OS was 8.6 weeks and 18.2 weeks respectively (Figure 1). The median PFS of patients with PR, SD and PD was 18, 9 and 4 weeks, respectively (*p* < 0.001). Patients with a better response had improved OS and PFS relative to patients with a worse response (*p* = 0.007 and *p* < 0.001, respectively, Figure 2).

### Ulcerative breast cancer

**Figure 3:**
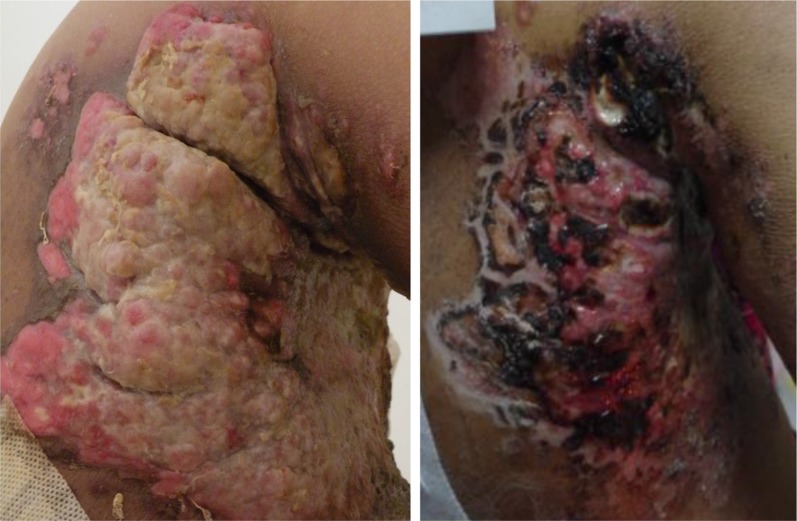
Carcinomatous ulcer in a 45-year-old woman with ER (+), PR (+) and HER2 (−) disease before (left) and after (right) sunitinib treatment Before treatment, the ulcer area was about 36 × 28 cm, significantly elevated from the skin by 1.2 cm, exuding, and not bleeding. After treatment, the area of the black scab increased. The tumor lesion elevated from the skin shrank significantly, and staxis was reduced.

Among the 9 patients with carcinomatous ulcers, 3 achieved PR and 4 achieved SD with tumor shrinkage; only 2 patients experienced PD. Among the 3 patients achieved PR, there was a 45-year-old woman with ER (+), PR (+) and HER2 (−) disease showed resistance to endocrine therapy, taxanes and anthracyclines. She was treated with sunitinib on a dosage of 37.5 mg/day in the eighth-line setting. After 1 month treatment, the area of black scab was increased. However, the tumor lesion elevated above the skin shrank significantly, and staxis was reduced (Figure 3 and Supplementary Materials).

### Immunohistochemistry findings

According to the results of IHC testing of 14 patients, there was no correlation between the clinical response to sunitinib and the expressions of VEGF, PDGFR, EGFR, or c-KIT (*p* = 0.689, 0.641, 0.126 and 0.495, respectively) (Table 2).

**Table 2:** Immunohistochemistry results of metastatic tumor in 14 patients

Patient No.	EGFR	PDGFR	VEGF	c-KIT	Clinical response
1	(−)	(+)	(+)	(−)	PR
2	(+)	(+)	(−)	(−)	PD
3	(+)	(+)	(−)	(+)	PD
4	(−)	(+)	(+)	(−)	PD
5	(+)	(−)	(+)	(−)	SD (improved)
6	(−)	(+)	(+)	(−)	PD
7	(−)	(+)	(+)	(−)	SD (improved)
8	(−)	(+)	(+)	(−)	SD
9	(−)	(+)	(+)	(−)	PR
10	(−)	(+)	(+)	(−)	PD
11	(−)	(+)	(+)	(−)	SD (improved)
12	(−)	(−)	(+)	(−)	PD
13	(−)	(+)	(+)	(−)	PD
14	(+)	(+)	(+)	(+)	PD

Abbreviations: EGFR, epidermal growth factor receptor; PD, progressive disease; PDGFR, platelet-derived growth factor receptor; PR, partial response; SD, stable disease; VEGF, vascular endothelial growth factor.

### Tolerability

The first 10 patients received 50 mg/day all experienced grade III/IV toxicity of neutrophil or platelet with median treatment time of 2 weeks. They had to reduce the dosage to 37.5 mg/day schedule. Subsequent 27 patients initially received 37.5 mg/day regimen, and the median treatment time was 3 weeks per cycle. For the target dose of 37.5 mg/day, a total of 14 (37.8%) patients experienced dosage reduction, and 12 (32.4%) patients required interruption of sunitinib due to adverse events.

5 patients were lost to follow-up and 3 patients died during the treatment. A total of 29 patients occured side effects. The major dose-limiting toxicities were myelosuppression and hypertension (Table 3). The most common adverse events included xanthochromia (100%), fatigue (82.8%), hypertension (34.5%), grade III/IV neutropenia (82.8%), and grade III/IV thrombocytopenia (79.3%) (Table 3). Other common adverse events included rash, cerebral hemorrhage and nausea.

**Table 3:** Toxicities/adverse events observed in 29 patients (*n*, %)

Toxicity/adverse event	Grade 0	Grade I	Grade II	Grade III	Grade IV
Xanthochromia	0 (0.0)	9 (31.0)	12 (41.4)	8 (27.6)	0 (0.0)
Fatigue	5 (17.2)	6 (20.7)	11 (37.9)	5 (17.2)	2 (6.9)
Hypertension	19 (65.5)	5 (17.2)	2 (6.9)	3 (10.3)	0 (0.0)
Subcutaneous hemorrhage	25 (86.2)	2 (6.9)	2 (6.9)	0 (0.0)	0 (0.0)
Cerebral hemorrhage	23 (79.3)	2 (6.9)	1 (3.4)	3 (10.3)	0 (0.0)
Anemia	25 (86.2)	1 (3.4)	3 (10.3)	0 (0.0)	0 (0.0)
Leucopenia	0 (0.0)	2 (6.9)	12 (41.4)	15 (51.7)	0 (0.0)
Neutropenia	0 (0.0)	2 (6.9)	3 (10.3)	24 (82.8)	0 (0.0)
Thrombocytopenia	0 (0.0)	3 (10.3)	3 (10.3)	22 (75.9)	1 (3.4)
Peripheral neuropathy	18 (62.1)	4 (13.8)	7 (24.1)	0 (0.0)	0 (0.0)
Hand-foot syndrome	23 (79.3)	4 (13.8)	2 (6.9)	0 (0.0)	0 (0.0)

## DISCUSSION

Investigation of efficacy of sunitinib in breast cancer stemmed from its significant antitumor effects on various solid malignant tumor cell lines, including breast cancer cell lines [6]. Its relatively low half maximal inhibitory concentration (IC_50_) for VEGFR2 suggests that it may exert an antiangiogenic effect on breast cancer [9]. A phase II clinical trial of sunitinib monotherapy to MBC was the first evaluation of its clinical efficacy in breast cancer [7]. A total of 64 patients previously failed on anthracycline and taxane drugs received sunitinib on a starting dosage of 50 mg daily in 6-week cycles with 4 weeks on followed by 2 weeks off. The results showed an overall ORR (objective response rate) of 11%, a clinical benefit rate (PR + SD ≥ 6 months) of 16%, a median PFS of 10 weeks, and a median OS of 38 weeks. Subsequently, four phase III clinical trials of sunitinib were conducted on a daily dosage of 37.5 mg to MBC patients [11–14]. The results showed that sunitinib as monotherapy or combined with chemotherapy failed to improve PFS and OS compared with other standard regimens for MBC. All studies to date that have investigated its efficacy in breast cancer showed overall ORR of 3–55% and median PFS of 2.0–8.6 months (Table 4).

**Table 4:** Summary of studies investigated the efficacy of sunitinib in the treatment of breast cancer

Publication	Phase	Patients and treatment stage	Total pts	Treatment regimens	Median f/u (m)	ORR (%)	Median PFS (m)	Median OS (m)
Yardley et al., 2015 [23]^#^	І/П	Locally advanced triple-negative breast cancer; neoadjuvant setting	54	S + weekly paclitaxel/carboplatin	23.1	pCR rate in 34 evaluable patients was 35%		
Burstein et al., 2008 [7]^#^	П	ABC pretreated with an anthracycline and a taxane; first- to fifth-line therapy	64	single-agent S	-	11	10 weeks	38 weeks
Wildiers et al. 2010 [24]	П	HER2-negative ABC patients achieved remissions induced by taxane-based chemotherapy; consolidation therapy	26 19	single-agent S no therapy	-	-	2.8 3.1 *p* = 0.173	- - *p* = 0.749
Curigliano et al., 2013 [25]^*^	П	Triple-negative ABC; first-, second- or third-line therapy	113 104	single-agent S standard of care chemotherapy	15.8 16.2	3 7 *p* = 0.962	2.0 2.7 *p* = 0.888	9.4 10.5 *p* = 0.839
Bachelot et al., 2014 [15]^#^	П	HER2-positive ABC; first- or second line	60	S + trastuzumab	24.4	37	6.4	NR
Niravath, et al., 2015 [26]^#^	П	Patients with central nervous system metastases received whole-brain radiotherapy concurrently with capecitabine	12	followed by S + capecitabine	-	0	4.7	10
Barrios et al. 2010 [11]	Ш	HER-2 negative ABC; first-, second- or third-line therapy	238 244	S Capecitabine	-	11 16 *p* = 0.109	2.8 4.2 *p* = 0.002	15.3 24.6 *p* = 0.350
Robert et al., 2011 [12]^*^	Ш	First-line for HER-2 negative ABC	242 243	S + Paclitaxel Bevacizumab + Paclitaxel	8.1	32 32 *p* = 0.525	7.4 9.2 *p* = 0.999	17.6 NR *p* = 0.996
Bergh et al., 2012 [13]^*^	Ш	First-line for HER-2 negative ABC	296 297	S + docetaxel docetaxel	18.0	55 42 *p* = 0.001	8.6 8.3 *p* = 0.265	24.8 25.5 *p* = 0.904
Crown et al., 2013 [14]^*^	Ш	Pretreated ABC (prior therapy with anthracycline and taxane); first-, second- or third-line therapy	221 221	S + Capecitabine Capecitabine	14.3	19 18 *p* = 0.490	5.5 5.9 *p* = 0.941	16.4 16.5 *p* = 0.484

All clinical trials are prospective, randomized, open-label studies. Publications of case reports and abstracts only are not listed. - indicates the specific number was not provided/applicable.

# represents single-arm trial.

* represents one-sided test.

Abbreviations: pts, patients; ABC, advanced breast cancer; S, sunitinib; ORR, objective response rate; PFS, progression-free survival; OS, overall survival; NR, not yet reached; m, month; f/u, follow-up.

We speculate the addition of sunitinib is hard to increase response due to the existing strong clinical efficacy generated by standard regimens and the incremental obvious side effects. Dose reduction or discontinuity of sunitinib will decrease effective drug intensity and then result in inferior response. Moreover, it should be noted that sunitinib was used as first-, second-, or third-line treatment in all phase III trials. Drug response is worse for patients received multiple-lines treatment than patients received less salvage treatment [15]. Therefore, the efficacy of sunitinib monotherapy for heavily pretreated patients is unknown. As we know, this population is excluded from almost all clinical studies, and they are recommended to receive palliative treatment by NCCN guidelines. It is cruel and unacceptable for most young, premenopausal patients in Asian counties in which more than 60% patients diagnosed with breast cancer are premenopausal women. Thus, we conducted this study and speculate that multitargeted agent which simultaneously inhibits multiple signaling might be appropriate and become a viable treatment choice for multi-resistant MBC. Our results showed that ORR was 18.8%, median PFS and OS were 8.6 and 18.2 weeks, respectively. The outcome was similar to that reported previously (Table 4).

Our study highlighted an interesting issue. Among the 9 patients who had carcinomatous ulcers, 3 achieved a PR and 4 achieved SD with tumor shrinkage. This suggested that sunitinib may be especially effective in carcinomatous ulcers. This effect might stem from either anti-vascular targeting or anti-cancer cell targeting or both. We speculate that the good outcomes may be due to a difference in biology of carcinomatous ulcers compared to visceral metastases and the accurate evaluation of the carcinomatous ulcers with superficial location. Due to the deep locations of the liver or lung metastases, we did not observe a reduction of the tumor volumes or maximal tumor diameters in these patients through conventional imaging tests such as CT or MRI. While the areas of ulcer lesions elevated above the skin were reduced and ulcer healing did occur, and tumor shrinkage was observed in most (77.8%) patients. Therefore, the effect of sunitinib on carcinomatous ulcers is promising and might be greater than present clinical evaluations. The visceral metastases are likely resistant to sunitinib due to very complicated mechanisms of resistance involving tumor vessels, angiogenic signaling pathways, tumor-stromal relationship and other poorly understood mechanisms [16, 17]. In this view, a comprehensive study focused on evaluation and mechanism of sunitinib efficacy in patients with troublesome carcinomatous ulcer is necessary.

The most frequently reported treatment-related side effects of sunitinib are xanthochromia, fatigue and gastrointestinal symptoms. And its main dose-limiting toxicities are myelosuppression and hypertension [18–22]. In this study, sunitinib treatment was initially conducted on a dosage of 50 mg/day. As none of first 10 patients could tolerate the dosage regimen, it was changed to a 37.5 mg/ day for the following patients. However, most patient was not able to tolerate continuous 37.5 mg/day regimen for more than 1 month. This might be due to the poor performance status of patients, the low body weight of Asian women, and the fact that they had failed on multiple regimens. In this regard, the use of a multitargeted agent with a dose-limiting toxicity of myelosuppression should be considered in the same light as the use of cytotoxic drugs. Therefore, we believe it is necessary to form a rational dosing strategy for sunitinib. For individual patients, either body surface area or body weight should be taken into the determination of optimum dosage.

In conclusion, this study in Chinese women with heavily pretreated refractory MBC indicated that sunitinib monotherapy has a modest therapeutic effect, especially for patient with troublesome carcinomatous ulcer. The treatment-related adverse events of sunitinib were manageable through dose adjustment. More appropriate patient population for sunitinib therapy and better administration of sunitinib regimen deserve further research.

## MATERIALS AND METHODS

### Patients

This is a prospective, open-label study approved by the Ethics Committee of Affiliated Hospital of Academy of Military Medical Sciences. A total of 37 multidrug-resistant MBC patients were enrolled to receive sunitinib alone from January 2010 to June 2011 in our institution.

Inclusion criteria included: (1) MBC diagnosed by pathology or cytology; (2) multidrug-resistant MBC defined as recurrent or metastatic tumor resistant to at least 3 previous salvage chemotherapy regimens, including trastuzumab if tumor was human epidermal growth factor receptor-2 (HER2)-positive and at least 1 endocrine agent if tumor was hormone receptor (HR)-positive; (3) age 18–70 years with a Karnofsky performance status (KPS) score ≥ 70 and anticipated survival of more than 3 months; (4) the presence of objectively evaluable tumors; (5) results of laboratory tests within normal reference ranges; and (6) the provision of written informed consent.

### Treatment

The first 10 patients enrolled received sunitinib on a full dosage of 50 mg orally once daily for 4 consecutive weeks followed by a 2-week off period. Because all 10 patients experienced grade III/IV hematologic toxicity, the study protocol was amended to reduce the dosage of sunitinib to 37.5 mg orally once daily with the same treatment/off schedule subsequently. Dosage adjustments allowed further dosage reduction (reduction with every 12.5 mg) depending on the severity of adverse events experienced, or discontinuation of sunitinib if any grade III/IV toxicities continued more than one week. Patient can not take sunitinib until severe toxicities decreased to grade I/II toxicities. If patients achieved stable disease (SD), complete response (CR) or partial response (PR), treatment cycle would be repeated until the present of either disease progression or intolerable toxicity.

### Response and toxicity criteria

Clinical responses were classified as CR, PR, SD or progressive disease (PD) according to the Response Evaluation Criteria in Solid Tumors (RECIST) version 1.1. Efficacy assessment included progression free survival (PFS), which was calculated from the date of sunitinib treatment to the date of confirmed PD or death. Overall survival (OS) analysis was calculated from the treatment of sunitinib to the date of breast cancer-related death or last follow-up. Tumor responses were assessed by objective imaging techniques such as computed tomography (CT) or magnetic resonance imaging (MRI). Clinical adverse events were graded according to the National Cancer Institute's Common Toxicity Criteria (NCI-CTC) version 3.0.

### Immunohistochemistry testing

We performed VEGF, PDGFR, EGFR and c-KIT test in metastatic tumor tissue using immunohistochemical (IHC) staining technique. The features of the immunoreaction were recorded on a semi-quantitative scale: the relative number of positive cells (0%, < 10%, 10–50% and > 50%) and the intensity of the reaction. The results were reported as positive if they were > 10% and negative if they were < 10% as per the SFDA guidelines. IHC staining for all the biomarkers was performed using a 1:250 dilution of the rabbit polyclonal antibody PV-6000 (ZSGB-BIO, CHN) with the EnVision detection system. The antigen retrieval method was not utilized. Appropriate positive and negative controls were used throughout the testing process.

### Statistical analysis

Data were analyzed using SPSS 22.0 software (SPSS, Inc., Chicago, IL, USA). Differences between values were examined using chi-square tests, and a *p*-value of ≤ 0.05 was considered statistically significant. The median PFS and OS was determined by the Kaplan-Meier method, and the survival curves were compared using log-rank test.

## SUPPLEMENTARY MATERIALS FIGURES


