# Supplementary material for: Sunitinib as salvage treatment including potent anti-tumor activity in carcinomatous ulcers for patients with multidrug-resistant metastatic breast cancer
Source: Oncotarget. 2016 Aug 5;7(36):57894–902. doi: 10.18632/oncotarget.11082 (PMC5295398; doi:10.18632/oncotarget.11082)
Supplement: Supplementary file 1 [file oncotarget-07-57894-s001.pdf]

Sunitinib as salvage treatment including potent anti-tumor activity in carcinomatous ulcers for patients with multidrug-resistant metastatic breast cancer

Supplementary Materials

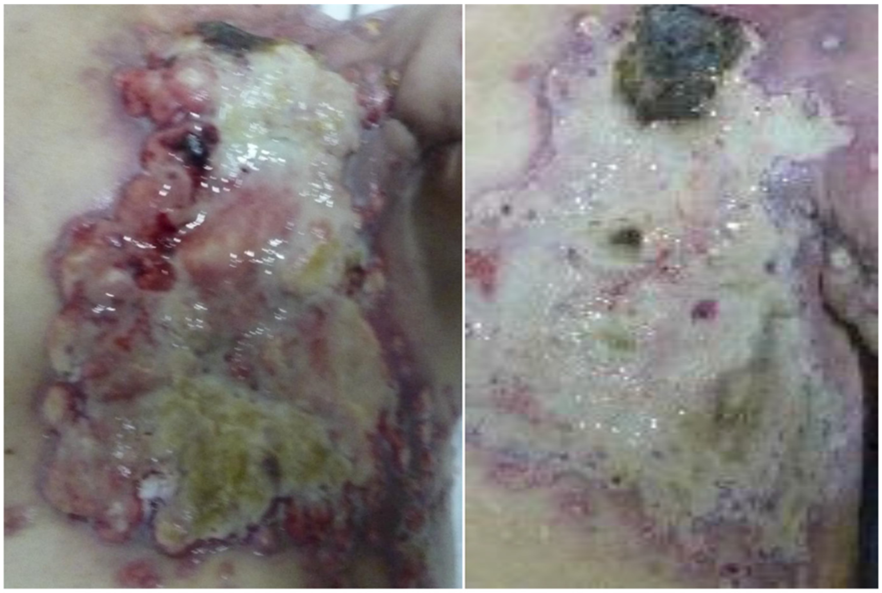

Pre-treatment During sunitinib treatment

Case 1: Pre-treatment: The chest wall ulcer with area of about 42 × 23 cm, with yellow-brown discharge, a small amount of bleeding, and stench, elevated from the chest wall. During sunitinib treatment: The secretion of chest wall was significantly reduced, odor was relief, and the range of left upper scab was increased.

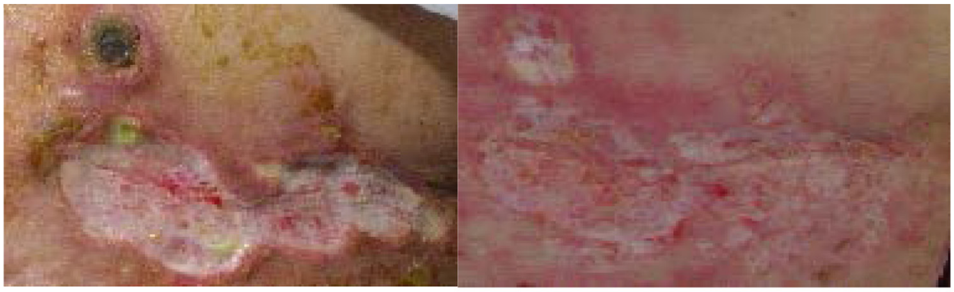

Pre-treatment During sunitinib treatment

Case 2: Pre-treatment: The area of chest wall sunken ulcer was about 10 × 4 cm. The ulcer had central scattered bleeding points, and it was surrounded by a number of miliary nodules protruding to the skin. During sunitinib treatment: The ulcer became flattened and pale, and the surrounded nodules significantly shrunk and even disappeared.

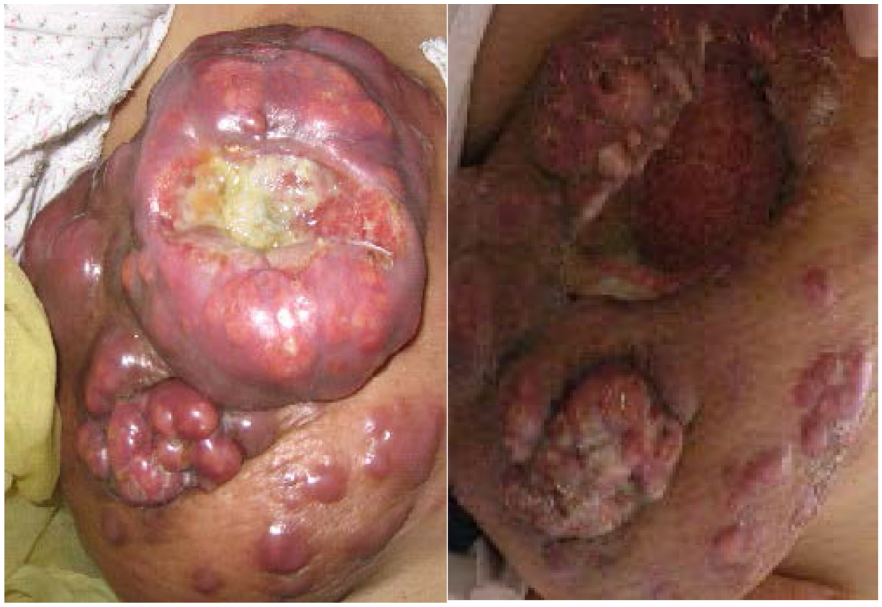

Pre-treatment During sunitinib treatment

Case 3: Pre-treatment: The patient had a huge tumor on her right breast with tumor volume of about 23 × 19 × 6 cm and a central deep cavity with depth of 3 cm. The tumor was oozing brown pus with bad odor and was surrounded by a number of tumors with various sizes. During sunitinib treatment: The primary mass has basically shrunk and appeared a large cavity with volume of about 12 × 9 × 8 cm. The exudate was reduced, odor weakened and the surrounding tumors shrunk.

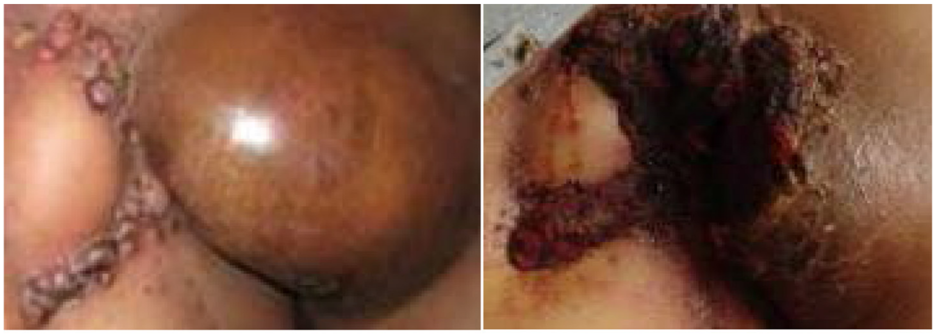

Pre-treatment During sunitinib treatment

Case 4: Pre-treatment: This patient had a huge mass in the left axillary with volume of about 19 × 17 × 9 cm. The tumor was hard and smooth. A number of miliary nodules were scattered in the chest wall. During sunitinib treatment: The great mass in left axillary appeared ulceration and a visible hole with volume of about 12 × 9 × 6 cm. Most of the ulceration had crusted with a little bleeding. Chest wall nodules also shrunk and became ulceration.
